# Pharmacologic Management of Intensive Care Unit Delirium and the Impact on the Duration of Delirium, Length of Intensive Care Unit Stay and 30-Day Mortality: A Network Meta-Analysis of Randomized-Control Trials

**DOI:** 10.7759/cureus.35843

**Published:** 2023-03-06

**Authors:** Muhammad Sohaib Afzal, Folajimi J Atunde, Rao Ahmed Yousaf, Shahid Ali, Namra Nasir, Gnana Deepthi Medarametla, Nazar Muhammad, Adil Amin

**Affiliations:** 1 Medicine, Louisiana State University Health Sciences Center, Shreveport, USA; 2 Neurology, NES Healthcare, Aylesbury, GBR; 3 Medicine, Faisalabad Medical University, Faisalabad, PAK; 4 Internal Medicine, Khyber Medical College, Peshawar, PAK; 5 Medicine, University of Health Sciences, Lahore, PAK; 6 Internal Medicine, AMMA Multispeciality Hospital, Thorur, IND; 7 Psychiatry, Nassau University Medical Center, New York City, USA; 8 Cardiology, Pakistan Navy Station (PNS) Shifa, Karachi, PAK

**Keywords:** meta-analysis, icu, dementia, atypical antipsychotic, typical antipsychotic

## Abstract

The present network meta-analysis was conducted to compare typical and atypical antipsychotics for the management of intensive care unit (ICU) delirium. The present meta-analysis was conducted in accordance with the Preferred Reporting Items for Systematic Reviews and Meta-Analyses (PRISMA) guidelines. Two investigators systematically searched electronic databases, including PubMed, EMBASE, and the Cochrane Library, for relevant studies in English from inception to February 15, 2023. The key terms used to search for relevant articles included “antipsychotic,” “delirium,” “randomized-controlled trials,” and “efficacy.” We used the term “randomized controlled trials (RCTs)” to limit the search to RCTs. The primary outcome was the duration of delirium in days. There were three predefined secondary outcomes included: mortality in 30 days, duration of mechanical ventilation in days, and length of ICU stay in days. A total of seven studies were included in the present meta-analysis. No significant difference was found between typical anti-psychotic, atypical anti-psychotic, and placebo in terms of duration of delirium, rate of mortality, duration of ICU stay, and duration of mechanical ventilation. In conclusion, this network meta-analysis comparing typical antipsychotic, atypical antipsychotic medications, and placebo on delirium in patients in the ICU did not find evidence that either typical or atypical antipsychotic medications led to a shorter duration of delirium. Patients who received treatment with typical or atypical antipsychotics and those who received a placebo had similar clinical outcomes, including mortality, length of stay in the ICU, and duration of ventilation.

## Introduction and background

Delirium is an acute disorder of cognition and consciousness that occurs in 60% to 80% of mechanically ventilated intensive care unit (ICU) patients [1]. Within this group of patients, acute brain dysfunction carries a significant risk of illness and death as it often leads to self-removal of breathing tubes, longer hospital stays, and higher mortality rates [2-4]. This kind of brain dysfunction is common in patients admitted to the ICU. The scale of the issue is estimated to increase in the coming years as the population ages and the demand for ICU care rises [5]. Moreover, it is believed that delirium in intensive care units results in healthcare expenditures between $6 and $20 billion every year in the United States [6]. Despite its evident clinical importance, delirium tends to be under-treated and underdiagnosed [7]. Thus, early identification, as well as efficient psychiatric treatment of delirium, is vital in the comprehensive care of ICU patients.

Delirium management includes ensuring safety with supportive or environmental interventions, treating and identifying the cause of delirium, and improving the functioning of patients [8]. Antipsychotic medications are often used for the treatment of delirium in the ICU [9,10]. The therapeutic effects of antipsychotics on delirium are not yet clear, but it is suggested that their effects may occur through the reduction of psychotic symptoms (known as positive symptoms in schizophrenia patients) or sedation. Two types of antipsychotic drugs are available: first-generation, also called typical antipsychotics (such as haloperidol and chlorpromazine), and second generation, also called atypical antipsychotics (such as quetiapine, olanzapine, and risperidone). Both types of antipsychotics block dopamine receptor pathways in the brain, but atypical antipsychotics also affect serotonin receptors. Both are effective in managing positive symptoms of schizophrenia (such as psychosis, hallucinations, and agitation), but atypical antipsychotics also improve negative symptoms, such as social and emotional withdrawal [11]. Atypical antipsychotic drugs like ziprasidone, risperidone, quetiapine, and olanzapine are also used for the management of delirium, and a placebo-controlled study has suggested an advantage [12]. On the other hand, the study conducted by Girard et al. [13] did not find any advantage of typical or atypical psychotic drugs over placebo in the duration of delirium in patients admitted to the ICU. This conflicting information is present in small studies and practice guidelines on the management of delirium in the ICU.

Antipsychotics are frequently the initial medication prescribed in clinical settings, even though there is contradictory evidence on their effectiveness, and reports indicate a greater likelihood of severe negative outcomes, particularly in vulnerable elderly patients. A previous meta-analysis compared both typical and atypical medications and was conducted in 2017 [11]. It includes both ICU and non-ICU patients, and therefore the findings could not be generalized to ICU patients as the interaction of antipsychotic medications is possible with different sedatives given to patients in the ICU. Therefore, the present meta-analysis focused on ICU patients. Moreover, due to a lack of studies comparing atypical and typical antipsychotic drugs, we performed a network meta-analysis comparing atypical, typical antipsychotic drugs and placebo simultaneously. As far as our knowledge is concerned, no previous meta-analysis has been conducted that compared all these options simultaneously in ICU patients. The aim of this meta-analysis was to compare typical and atypical antipsychotics for the management of intensive care unit (ICU) delirium.

## Review

Methodology

The present meta-analysis was conducted in accordance with the Preferred Reporting Items for Systematic Reviews and Meta-Analyses (PRISMA) guidelines.

Search Strategy and Study Selection

Two investigators systematically searched electronic databases, including PubMed, EMBASE, and the Cochrane Library, for relevant studies in English from inception to February 15, 2023. The key terms used to search for relevant articles included “antipsychotic,” “delirium," “randomized-controlled trials,” and “efficacy.” We used the term “randomized controlled trials (RCTs)” to limit the search to RCTs. The reference lists of all selected studies were manually screened for other potentially eligible studies.

All eligible studies were screened using standard forms. Firstly, eligible studies were reviewed using titles and abstracts by two authors independently to identify all potentially relevant papers. Moreover, the same two investigators reviewed the full text of all eligible studies to assess detailed inclusion and exclusion criteria. Any disagreement between the two authors in the process of study selection was resolved via discussion, or the involvement of a third investigator if required.

Eligibility Criteria

We included all RCTs that compared typical antipsychotics and atypical antipsychotics with each other or with a placebo for the treatment of delirium (hypoactive and hyperactive delirium) in ICU patients. We included studies that were conducted in the adult population (>18 years). We excluded studies that enrolled non-ICU patients. Additionally, we excluded observational studies, case reports, reviews, and non-randomized trials. We also excluded studies published in a language other than English.

Data Extraction and Quality Assessment

Two authors independently extracted data from relevant studies using a pre-designed data collection form developed in Microsoft Excel. Extracted data included the principal author's name, year of publication, the region where the study was conducted, groups, sample size, mean age in years, number of males, and outcomes.

The Cochrane risk of bias assessment tool was used to assess the methodological quality of all included studies. Two authors assessed the study quality independently, and any disagreement between the two authors was resolved by discussion or via consensus with the principal investigator.

Outcomes

The primary outcome was the duration of delirium in days. There were three predefined secondary outcomes included: mortality in 30 days, duration of mechanical ventilation in days, and length of ICU stay in days.

Statistical Analysis

A network meta-analysis was conducted using STATA version 16.0 based on the Bayesian framework model. To analyze the impact of treatment on dichotomous variables, the odd ratio (OR) was calculated along with their 95% confidence interval (CI), while for continuous variables, we calculated the mean difference (MD) with 95% CI. Rank plots based on cumulative probabilities were performed for different outcomes to identify the best treatment. Inconsistency was evaluated by node-splitting analysis and a loop-specific approach. We did not assess publication bias because of the limited number of studies.

Results

**Figure 1:**
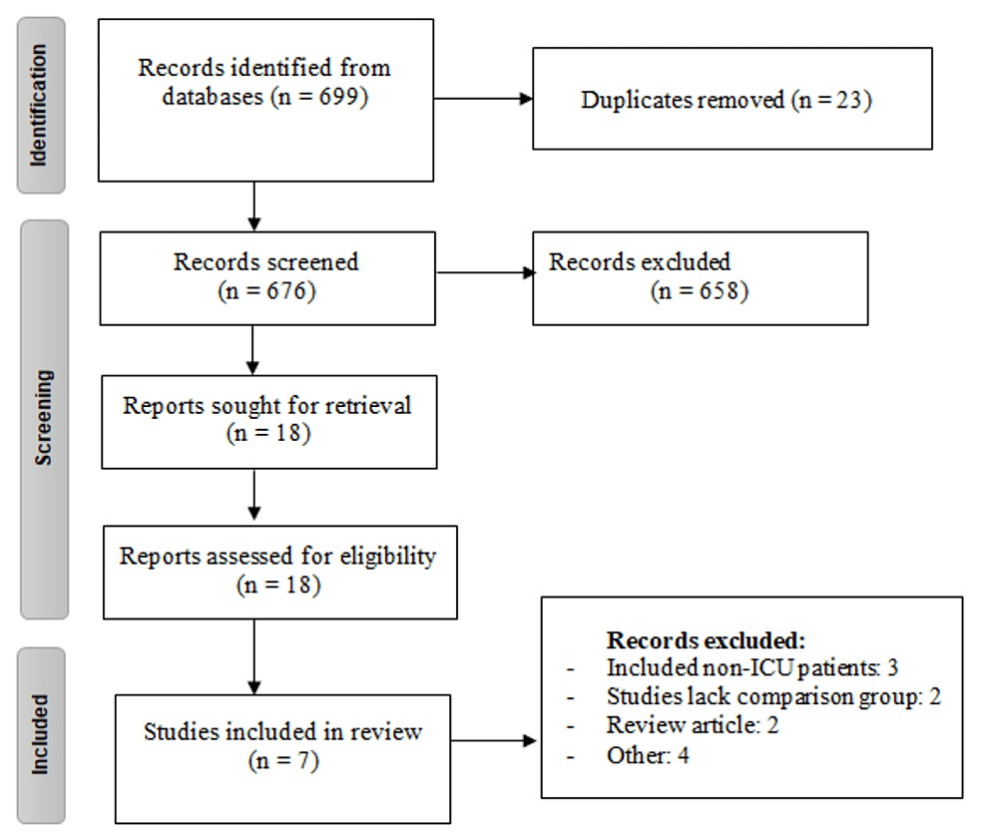
PRISMA flowchart of selection of studies.

**Figure 2:**
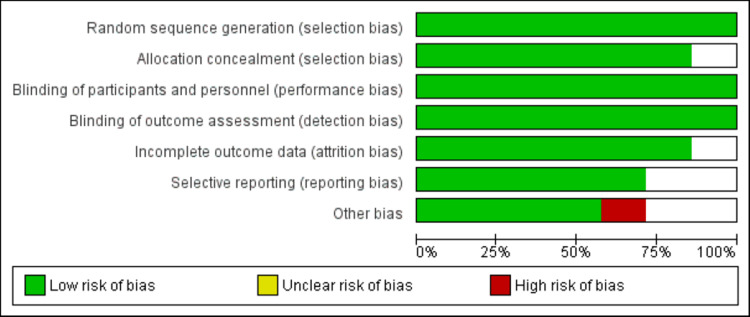
Risk of bias assessment.

Figure 1 depicts the PRISMA flowchart outlining the process of study selection. A total of 699 studies were identified from electronic databases. After removing duplicates, we assessed the title and abstract of 676 studies. During this process, 658 studies were excluded based on screening of titles and abstracts. Upon evaluation of the full-text, 11 studies were further excluded for various reasons, as shown in Figure 1. Ultimately, a total of seven studies were included in the present meta-analysis. Table 1 presents the characteristics of all included studies. Of the 7 RCTs, four were conducted in the United States. All studies that assessed typical antipsychotic medications used haloperidol, while two studies used ziprasidone and two used quetiapine as atypical antipsychotic medications. The mean age of patients in the included studies ranged from 53.7 to 74.2 years. In all the included studies, the majority of participants were male. Figure 2 shows the risk of bias assessment. Overall risk of bias was low.

**Table 1:** Characteristics of the included studies

Author Name	Year	Country	Groups	Sample Size	Groups	Mean age (Years)	Males (%)
Al-Qadheeb et al. [14]	2016	United States	Typical	34	Haloperidol	60.5	55.9
			Placebo	34	Placebo		
Boogaard et al. [15]	2018	Netherlands	Typical	732	Haloperidol	66.9	62.1
			Placebo	707	Placebo		
Devlin et al. [12]	2010	United States	Atypical	18	Quetiapine	63	55.6
			Placebo	18	Placebo		
Garg et al. [5]	2022	India	Typical	15	Halopreridol	57.7	55.6
			Atypical	15	Quetiapine		
			Placebo	15	Placebo		
Girard et al. [13]	2010	United States	Typical	35	Haloperidol	53.7	62.4
			Atypical	30	Ziprasidone		
			Placebo	36	Placebo		
Girard et al. [16]	2018	United States	Typical	192	Haloperidol	60.5	57.1
			Atypical	190	Ziprasidone		
			Placebo	184	Placebo		
Wang et al. [17]	2012	China	Typical	229	Haloperidol	74.2	63
			Placebo	228	Placebo		

Clinical outcomes

Duration of Delirium (Days)

**Figure 3:**
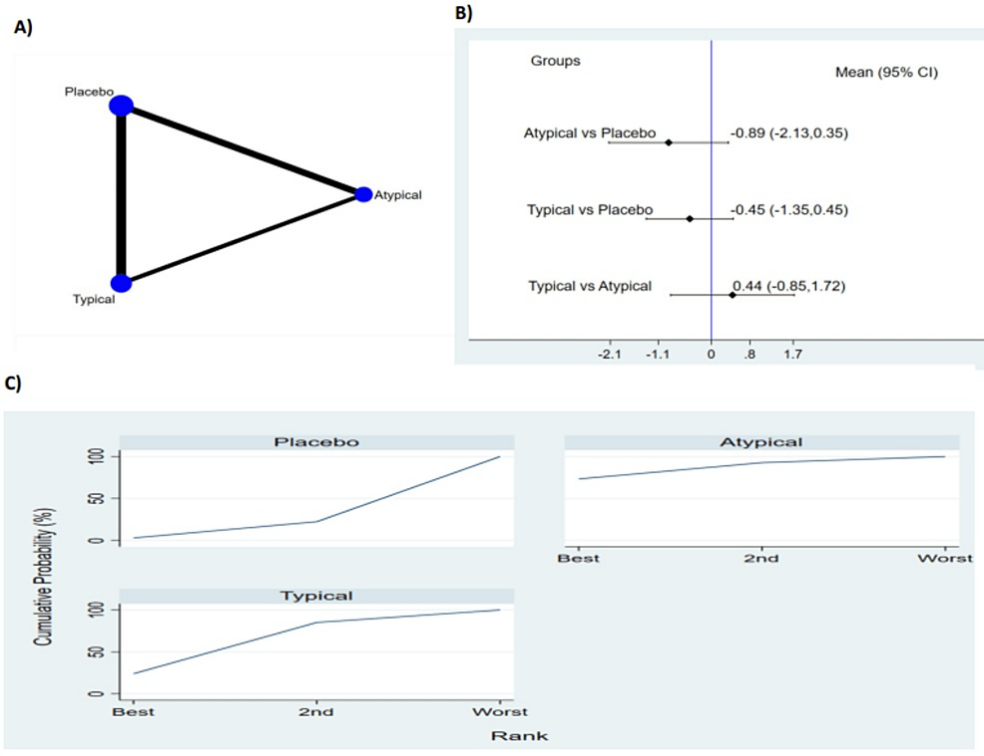
Results of duration of delirium (A) network plot of eligible comparison. The size of node reflects the sample size, and the width of the line shows the number of studies being compared. (B) The forest plot of network analysis. The black diamond shows the combined mean difference. Mean difference > 0 shows the delirium duration is higher in the former group compared to the latter group. (C) The cumulative ranking curve of delirium duration. CI: Confidence interval

Five studies reported duration of delirium (Figure 3A). The pooled results no significant difference between typical antipsychotic, atypical antipsychotic medications and placebo in terms of duration of delirium as shown in Figure 3B. The probability based ranking result is shown in Figure 3C. Results of probability-based ranking showed that atypical antipsychotic ranked first, typical psychotic ranked second and the placebo ranked third. In this result, the first rank had the lowest duration of delirium.

All-Cause Mortality

**Figure 4:**
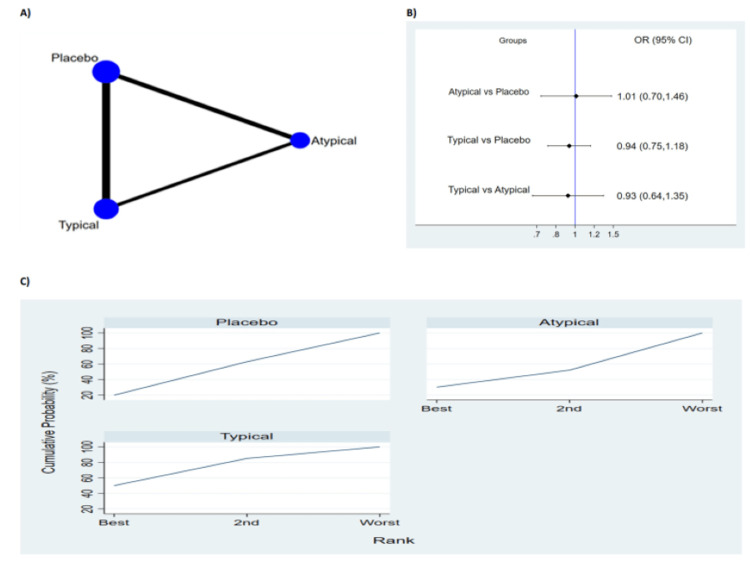
Results of all cause-mortality (A) Network plot of eligible comparison. The size of node reflects the sample size and the width of the line shows the number of studies being compared. (B) The forest plot of network analysis. The black diamond shows the combined OR. OR>1 shows the mortality is higher in the former group compared to the latter group. (C) The cumulative ranking curve of mortality. OR: Odds ratio; CI: Confidence interval

Seven studies with 2712 patients reported mortality. The network geometry is shown in Figure 4A. The pooled meta-analysis results showed no significant difference between typical antipsychotic, atypical antipsychotic medications and placebo as shown in Figure 4B. The probability-based ranking result is shown in Figure 4C. Results of Bayesian analysis showed that typical psychotic ranked first, atypical psychotic ranked second and the placebo ranked third in terms of prevention of all-cause mortality (Figure 3B). In this result, the first rank had the lowest proportion of mortality.

Length of ICU Stay in Days

**Figure 5:**
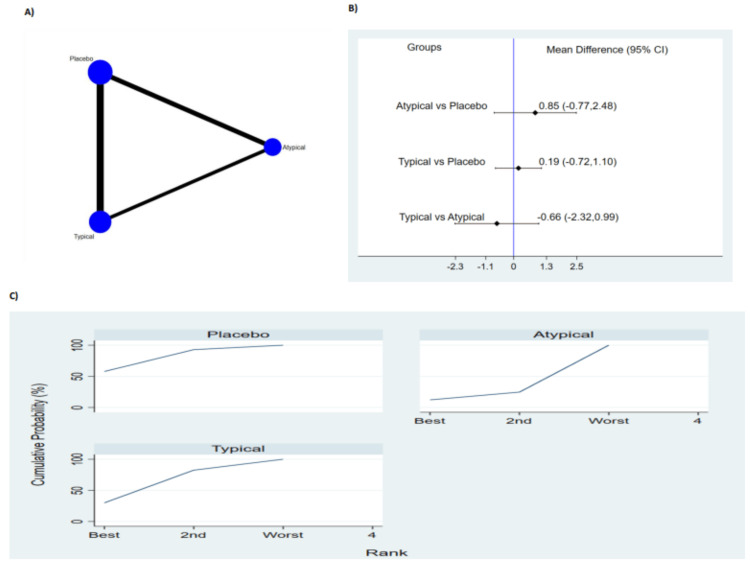
Results of duration of ICU stay (A) Network plot of eligible comparison. The size of node reflects the sample size and the width of the line shows the number of studies being compared. (B) The forest plot of network analysis. The black diamond shows the combined mean difference. Mean difference >0 shows the ICU stay is higher in the former group compared to the latter group. (C) The cumulative ranking curve of ICU stay. CI: Confidence interval

Seven studies involving 2,712 patients reported duration of ICU stay in days (Figure 5A). The pooled results showed no significant difference between typical antipsychotic, atypical antipsychotic medications and placebo in relation to duration of ICU stay (Figure 5B). The probability-based ranking result is shown in Figure 5C. Results of probability based ranking shows that placebo ranked first, typical antipsychotic ranked second and atypical antipsychotic ranked third. In this result, the first rank had the highest proportion had the lowest ICU stay.

Duration of Mechanical Ventilation in Days

**Figure 6:**
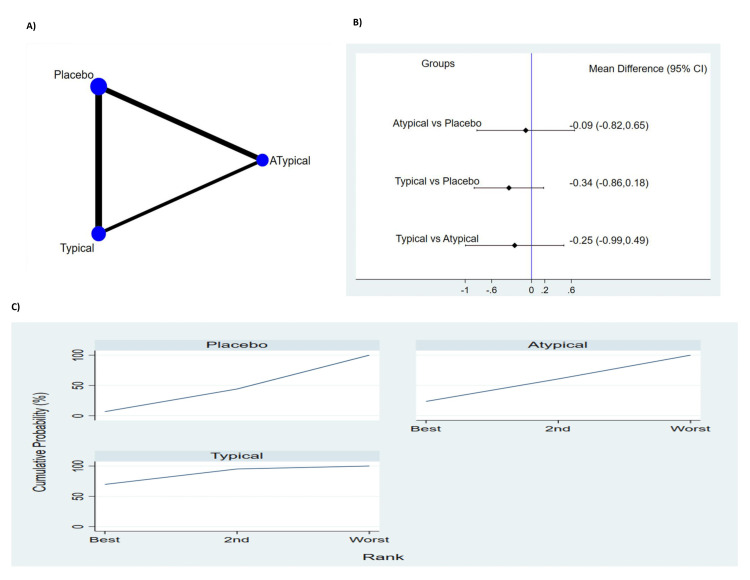
Results of duration of mechanical ventilation (A) Network plot of eligible comparison. The size of node reflects the sample size and the width of the line shows the number of studies being compared. (B) The forest plot of network analysis. The black diamond shows the combined mean difference. Mean difference >0 shows the duration of mechanical ventilation is higher in the former group compared to the latter group. (C) The cumulative ranking curve of duration of mechanical ventilation. CI: Confidence interval

Five studies reported duration of mechanical ventilation in days (Figure 6A). The pooled results showed no significant difference between typical antipsychotic, atypical antipsychotic medications and placebo in relation to duration of mechanical ventilation (Figure 6B). The probability-based ranking result is shown in Figure 6C. Results of probability based ranking shows that typical antipsychotic ranked first, atypical antipsychotic ranked second and placebo ranked third. In this result, the first rank had the lowest mechanical ventilation in days.

Discussion

This network meta-analysis compared typical antipsychotic, atypical antipsychotic medications, and placebo in relation to delirium in patients in the intensive care unit (ICU). The present meta-analysis included seven RCTs involving 2,712 patients. To our knowledge, it is the first network meta-analysis comparing antipsychotic, atypical antipsychotic medications, and placebo in ICU patients with delirium. Traditional meta-analysis only permits the direct comparison of two treatment options that have been evaluated head-to-head. However, recently conducted RCTs have included three groups, including antipsychotic, atypical antipsychotic medications, and placebo. Therefore, a network meta-analysis has been conducted that compares all treatment groups simultaneously within a single framework and ranks treatment options based on efficacy.

In this network meta-analysis, no evidence was reported that either typical or atypical antipsychotic medications led to a shorter duration of delirium. Patients who received treatment with typical or atypical antipsychotic medications and those who received a placebo had similar clinical outcomes, including mortality, length of stay in the ICU, and duration of ventilation. Previous studies have reported that the efficacy of atypical antipsychotic medications is not different from haloperidol in the treatment of delirium [18-20].

One possible reason why we did not find evidence that the use of atypical or typical antipsychotic medications resulted in fewer days with delirium compared to placebo in ICU patients is that these drugs target increased dopamine signalling in the brain, which may not be a major factor in causing delirium during critical illness. It is also possible that there are several different mechanisms that cause delirium in critically ill patients, and sedation with γ-aminobutyric acid agonists is a common risk factor for delirium during critical illness [21].

Our principal findings were consistent with a recent review conducted by Neufeld et al. [22]. They found that antipsychotic medications are not effective for the treatment or prevention of delirium in hospitalized patients. However, our meta-analysis included two recently conducted RCTs. Secondly, our review included studies that were exclusively conducted on ICU patients. Moreover, the review conducted by Neufeld et al. was a traditional meta-analysis and did not consider all three groups simultaneously [22].

If the underlying physical causes of delirium are treated and risk factors for delirium are reduced, antipsychotic medications can be helpful. In non-ICU settings, non-drug interventions have been tested to reduce the risk of delirium, but there is no evidence of their effectiveness in the ICU [23]. Among pharmacological treatments for delirium in the ICU, sedating patients with the drug dexmedetomidine, which activates a specific receptor in the brain, has been shown to be more effective at reducing the duration of delirium and coma compared to using benzodiazepines for sedation [24].

Although no significant difference was found in the efficiency of typical antipsychotic, atypical antipsychotic medications, and placebo, the results of the cumulative probability graph showed that atypical antipsychotic medication ranked as the most effective option in terms of less duration of delirium, followed by typical antipsychotic medication and placebo, but no significant differences were found among the treatment options. However, considering the number of studies included in this meta-analysis, the findings need to be interpreted with caution. In the future, more prospective studies are needed in ICU patients to develop the best possible treatment guidelines for the management of delirium in this population.

Study limitations

The current meta-analysis has certain limitations. Firstly, only seven studies were included in the present meta-analysis and number of studies comparing atypical and typical antipsychotic medications with each other. Secondly, due to less number of studies, we were not able to assess the publication bias. In the present meta-analysis, we did not analyze the efficiency of sedatives on the clinical outcomes on account of absence of patient level data. The use of certain sedatives like benzodiazepines, may have reduced the threshold of occurrence of delirium, while dexmedetomidine along with light sedation may add on additional benefit for prevention of delirium.

## Conclusions

In conclusion, this network meta-analysis comparing typical antipsychotic, atypical antipsychotic medications, and placebo on delirium in patients in the ICU did not find evidence that either typical or atypical antipsychotic medications led to a shorter duration of delirium. Patients who received treatment with typical or atypical antipsychotics and those who received a placebo had similar clinical outcomes, including mortality, length of stay in the ICU, and duration of ventilation. However, only seven studies were included in this meta-analysis and only three studies compared typical, atypical antipsychotics and placebo simultaneously. In the future, large RCTs are needed including a larger sample size in the ICU setting to assess the impact of different treatments in ICU delirium. 
